# Serology reveals comparable patterns in the transmission intensities of *Plasmodium falciparum* and *Plasmodium vivax* in Langkat district, North Sumatera Province, Indonesia

**DOI:** 10.3389/fcimb.2025.1504741

**Published:** 2025-02-17

**Authors:** Inke Nadia Diniyanti Lubis, Irbah Rea Alvieda Nainggolan, Meliani Meliani, Beby Syofiani Hasibuan, Kumuthamalar Sangaran, Luqman Samsudin, Sriwipa Chuangchaiya, Paul Cliff Simon Divis, Ranti Permatasari, Zulkarnain Md Idris

**Affiliations:** ^1^ Faculty of Medicine, Universitas Sumatera Utara, Medan, Indonesia; ^2^ Department of Parasitology and Medical Entomology, Faculty of Medicine, Universiti Kebangsaan Malaysia, Kuala Lumpur, Malaysia; ^3^ National Public Health Laboratory, Ministry of Health, Sungai Buloh, Selangor, Malaysia; ^4^ Vector-Borne Disease Unit, Lipis District Health Office, Kuala Lipis, Pahang, Malaysia; ^5^ Department of Community Health, Faculty of Public Health, Kasetsart University, Sakon Nakhon, Thailand; ^6^ Malaria Research Centre, Faculty of Medicine and Health Sciences, Universiti Malaysia Sarawak, Kota Samarahan, Sarawak, Malaysia

**Keywords:** malaria, *P. falciparum*, *P. vivax*, serology, transmission, Indonesia

## Abstract

**Introduction:**

The incidence of malaria in Indonesia has declined significantly over the last few decades. Thus, a demand for more sensitive techniques to describe low levels of transmission in the country is important. This study was conducted to evaluate antibody response to *Plasmodium falciparum* and *Plasmodium vivax* in an area nearing elimination in North Sumatera Province, Indonesia.

**Methods:**

A cross-sectional survey was conducted in Langkat district, North Sumatera Province, in June 2019. Basic demographic data and filter paper blood spots were collected from 339 participants. Antibody responses to two *P. falciparum* (PfAMA-1 and PfMSP-1_19_) and two *P. vivax* (PvAMA-1 and PvMSP-1_19_) antigens were measured using indirect enzyme-linked immunosorbent assay (ELISA). Seroconversion rates (SCR) were estimated by fitting a simple reversible catalytic model to seroprevalence data for each antibody. Multiple logistic regression was used to investigate factors associated with exposure.

**Results:**

The overall malaria seroprevalence was 10.6% for PfAMA-1, 13% for PfMSP-1_19_, 18.6% for PvAMA-1, and 7.4% for PvMSP-1_19_. Seropositive individuals for *P. falciparum* (PfAMA-1/PfMSP-1_19_) and *P. vivax* (PvAMA-1/PvMSP-1_19_) were similar at 20.7%, with no significant differences observed between age groups (p > 0.05). Based on the reversible catalytic model, the calculated SCRs indicated a higher level of *P. falciparum* transmission than *P. vivax* using all tested antigens. In the adjusted model, only spending nights in the forest was associated with *P. vivax* seropositivity (odd ratio: 3.93, p < 0.001).

**Conclusion:**

The analysis of community-based serological data helps describe the similar levels of *P. falciparum* and *P. vivax* transmission in the Langkat district. The use of a serological approach enhances the detection of past exposure, aiding in the identification of epidemiological risk factors and malaria surveillance in low transmission settings in Indonesia.

## Introduction

Malaria remains a significant global health concern, particularly in tropical and subtropical countries. Indonesia, one of nine malaria-endemic countries in tropical Southeast Asia, has set a goal to eliminate malaria by 2030 (WHO, 2015). The country has made remarkable progress in malaria control, reducing the incidence from 1.8 million cases in 2011 to 811,636 cases in 2021 (WHO, 2023). In 2022, it was estimated that only 6.4% of Indonesia’s population was at high risk of malaria with 8.2% still living in active foci areas (WHO, 2023). As malaria-endemic areas in Indonesia shrink and become more localized, ongoing efforts to control and monitor the disease are crucial to containing its transmission.

Measuring malaria transmission patterns is important for effectively targeting control strategies and evaluating their impact after implementation. One method to estimate transmission involves assessing the malaria-specific immune responses in local populations, which serve as indicators of exposure to infection (van den Hoogen and Drakeley, 2017). Serological markers offer an alternative to traditional surveillance methods, allowing for the effective measurement of malaria transmission through the detection of antimalarial antibodies developed in response to the parasite antigens (Zakeri et al., 2016; Idris et al., 2017a). In low malaria transmission settings, this validated approach not only serves as a proxy to conventional methods but also provides greater sensitivity and reliability (van den Hoogen and Drakeley, 2017). As such, it is a valuable tool for guiding tailored malaria control programs and monitoring changes in transmission following intervention (Cook et al., 2010; Dewasurendra et al., 2017; Idris et al., 2017b; Macalinao et al., 2023). Numerous studies conducted in low-endemicity regions have demonstrated positive outcomes in using seroepidemiological analysis in determining the malaria burden, leading to improved public health policy planning (Rosas-Aguirre et al., 2013; Zakeri et al., 2016; Idris et al., 2017a; Keffale et al., 2019; Rahim et al., 2022; Rahim et al., 2023; Pinedo-Cancino et al., 2024).

Investigating the application of serological metrics in order to understand the historical patterns of malaria transmission in a population is essential in Indonesia. Whilst several seroepidemiological studies have been conducted in provinces in Indonesia with a very low prevalence of *Plasmodium falciparum* and *Plasmodium vivax* namely Central Java (Bretscher et al., 2013), Lampung (Supargiyono et al., 2013), Aceh (Surendra et al., 2019) and Yogyakarta (Surendra et al., 2020), no such study has been carried out in North Sumatera Province where the prevalence of *Plasmodium knowlesi* and multispecies of human malaria have been reported (Lubis et al., 2017; Nainggolan et al., 2022). In the present study, antibody responses to *P. falciparum* and *P. vivax* blood-stage antigens apical membrane antigen 1 (AMA-1), and merozoite surface antigen-1_19_ (MSP-1_19_) were measured to assess malaria exposure and transmission in North Sumatera Province.

## Materials and methods

### Ethics statement

The study was conducted following the Declaration of Helsinki and was approved by the Ethics Committee of the Faculty of Medicine, Universitas Sumatera Utara (No. 179/TGL/KEPK FK-USU-RSUPHAM/2019). Participants were sensitized to the study objectives and procedures by the local health district personnel for the study participation.

### Study area

**Figure 1:**
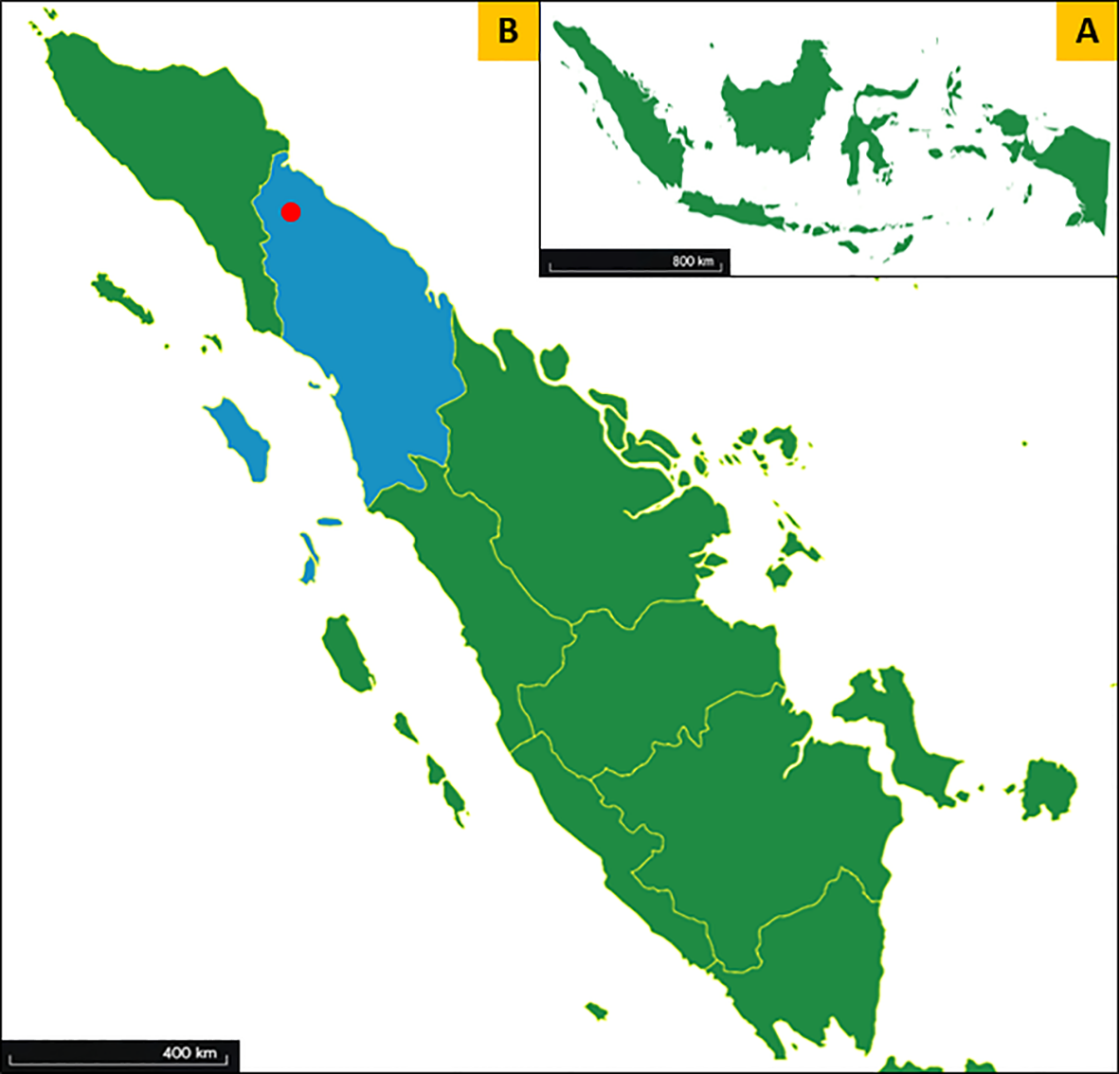
Map of the study area. **(A)** Map of the Republic of Indonesia. **(B)** Map of Sumatera Island in Indonesia showing the location of the study area (red circle) within Langkat district, North Sumatera Province (blue).

A community-based cross-sectional survey using a convenience sampling strategy was conducted in Langkat district, North Sumatera Province, Indonesia, in June 2019 (**Figure 1**). The dominant ethnic group in the study area is Batak Karo; Karo dialect is primarily spoken, as well as the national language of Indonesia (Nainggolan et al., 2022). Langkat district covers 6,263 km² with an altitude ranging from 4 to 105 meters above sea level. In 2019, the population of Langkat was estimated at 1,041,775 inhabitants (Pemerintah kabupaten Langkat, 2014). All villages share similar environmental characteristics, as they are located in the middle of the forest, with forestry and agriculture being the primary economic activities. These forest activities by the local population could potentially increase malaria transmission to outdoor-biting and forest-dwelling mosquitoes. This study is the first to assess the seroprevalence of malaria in the area, building on previous knowledge of a very low malaria prevalence, including a 0.3% microscopic infection rate, zero rapid diagnostic test (RDT)-positive cases, and a 0.9% submicroscopic infection rate in the population (Nainggolan et al., 2022).

### Sample collection

The sample size for study participation was calculated using Cochran’s formula: N = z²p(1 – p)/e², where z is the 95% confidence interval (z-value of 1.96), p is the expected prevalence of malaria (11.4% from a previous study by Surendra et al (Surendra et al., 2019)), and e is the allowed error margin (5%). Based on these considerations, a minimum size of 155 participants was calculated. The study protocol was explained to participants, and informed consent was documented, with provisions for illiterate participants and those under 18 years requiring parental or guardian consent. Participants were informed of their right to withdraw from the study at any time without prejudice. Village leaders and household heads were informed about the study’s objectives and procedures and asked to invite residents to the survey point. Inclusion criteria were individuals over 6 months old who had lived in the area for at least 6 months and consented to participate, while exclusion criteria included physically or mentally unfit individuals and incomplete examination data.

A standardized questionnaire was used to collect sociodemographic information from each participant. Finger prick blood samples were collected to prepare for dried blood spots (DBSs) on Whatman 3MM filter paper (Whatman, UK). Axillary body temperature was determined using a digital thermometer and fever was defined as a temperature exceeding 37.5°C. Hemoglobin (Hb) level was measured with the HemoCue Hb 201 analyzer (HemoCue, Sweden). Anemia was defined based on the concentrations of Hb in the blood according to WHO criteria (WHO, 2011). The DBS samples were air-dried and stored individually in sealed plastic bags. All DBS samples were transported under cold conditions to the laboratory in the Faculty of Medicine, Universitas Sumatera Utara, Medan, and stored at −20°C until further processing.

### Serological assay

A serum elution from 6-mm diameter DBS punches was used as previously described (Idris et al., 2017a; Idris et al., 2017b; Rahim et al., 2022; Rahim et al., 2023). Briefly, proper elution of plasma from DBSs (i.e. equivalent to a 1:200 dilution of serum) was assessed by the color change of the spots (to white) as well as the elution (to red/brown) after soaking them for 1–2 nights in reconstitution solution at ambient temperature while on a horizontal shaker. If blood spots did not change color, still retaining the brownish blood color, spots were soaked further until a color change was observed or excluded from analyses as reported before (Corran et al., 2008). Antibody responses (immunoglobulin G) against apical membrane antigen-1 or the 19-kDa fragment of merozoite surface protein-1 for *P. falciparum* (PfAMA-1 and PfMSP-1_19_, respectively) and *P. vivax* (PvAMA-1 and PvMSP-1_19_) were tested using enzyme-linked immunosorbent assay (ELISA) as previously described (Idris et al., 2017a; Idris et al., 2017b; Rahim et al., 2022; Rahim et al., 2023). Briefly, sera from the reconstituted blood spot samples were added in duplicate at a final concentration of 1:1,000 for MSP-1_19_ and 1:2,000 for AMA-1. In addition, four wells of malaria-naïve Malaysians sera as negative controls and a fivefold dilution series (starting at 1:100 for AMA-1 and 1:50 for MSP-1_19_) of a hyper-immune plasma pool (n = 15) were added per plate. Optical density (OD) values were measured at 450 nm with a Multiskan Go ELISA microplate reader (Thermo Scientific, USA).

### Data analysis

The gathered data were compiled into a Microsoft Excel spreadsheet and cross-checked for errors, with all further statistical analyses conducted in STATA version 13.1 (StataCorp, TX, USA). Continuous variables were presented using the median and interquartile range (IQR), while categorical variables were described using frequencies and percentages. Differences in proportions were tested using the chi-squared test or Fisher’s exact test. Duplicate ODs per individual were averaged, adjusted for background reactivity, and normalized against the positive control curve as previously described to adjust for plate variation (Corran et al., 2008). Seropositivity thresholds for separate antigens were calculated using a finite mixture model (Stewart et al., 2009), defining individuals as seropositive when their adjusted OD value exceeded the mean of the lower Gaussian distribution plus three times the standard deviation. The reversible catalytic model was employed to define the seroconversion rate (SCR) and plot corresponding seroconversion curves while fitting age-adjusted seropositivity to *P. falciparum* or *P. vivax* using maximum likelihood (Drakeley et al., 2005). Infants under one year of age were excluded from the reversible catalytic model to eliminate the influence of maternally derived antibodies (Drakeley et al., 2005). Factors associated with *P. falciparum* and *P. vivax* seropositivities were determined independently for each site using generalized estimating equations, adjusting for correlation between observations from the same variables. Variables significant at p < 0.10 in the univariate analyses were incorporated into the multivariate model and retained in the final model if their association with immune responses was statistically significant at p < 0.05.

## Results

### Characteristics of the study population

A total of 339 individuals were sampled during a cross-sectional survey in Langkat district, Sumatera Utara Province, Indonesia, in June 2019 (**Table 1**). The median age was 39 years old (IQR 19-54) and were mostly female (68.1%). Individuals with fever and anemia at enrolment accounted for 0.6% and 36.9%, respectively. A total of 36.9% of the study participants reported having at least one bed net in their house, resulting in an overall usage of 42.2%. The majority of the participants were forest-goers (58.7%) and live within the forest fringes (86.1%). Approximately 35.7% of the study participants reported spending a night in the forest within the last 2 weeks and only 0.6% reported a history of malaria over the past 3 months.

**Table 1:** General characteristics of the study population in Langkat district, North Sumatera Province, Indonesia in 2019.

Demographic data	% (95% CI)
Sample size, n	339
Median age (IQR)	39 (19-54)
<15	24.5 (19.9-29.4)
16 – 30	13.6 (10.1-17.7)
31 – 45	23 (18.6-27.9)
>45	38.9 (33.7-44.4)
Female	68.1 (62.9-73.1)
Fever^a^	0.6 (0.1-2.1)
Anemia^b^	36.9 (31.7-42.3)
ITN ownership	30.7 (25.8-35.9)
Sleep under ITN every night	42.2 (36.9-47.6)
Forest-goers	58.7 (53.3-63.9)
Live within forest fringes	86.1 (81.9-89.6)
Spend nights at the forest^c^	35.7 (0.31-0.41)
History of malaria^d^	0.6 (0.1-2.1)

IQR, Interquartile range; CI, Confident interval; ITN, Insecticide-treated net.

a Defined as a temperature exceeding 37.5°C.

b Based on the concentrations of Hb in the blood according to WHO criteria.

c Within the last 2 weeks.

d Reported history of malaria over the past 3 months; confirmed by local health records.

### Antibody response and seroprevalence

**Table 2** shows the age-specific seroprevalence and seroconversion rates of *P. falciparum* and *P. vivax* antigens among participants. Overall, malaria seroprevalence was 10.6% for PfAMA-1, 13% for PfMSP-1_19_, 18.6% for PvAMA-1, and 7.4% for PvMSP-1_19_. For all parasite antigens, no significant difference was observed in the proportion of seropositive individuals with increased age (all p > 0.05). Between species-specific antigens and age groups, the proportion of seropositive individuals was significantly higher for PvAMA-1 compared to PvMSP-1_19_ (all p < 0.05), except for 16-30 years (p = 0.354). Furthermore, the proportion of participants who were seropositive for either *P. falciparum* antigens (PfAMA-1/PfMSP-1_19_) or *P. vivax* antigens (PvAMA-1/PvMSP-1_19_) were similar at 20.7%, with no significant differences observed between age groups (p > 0.05).

**Table 2:** Age-specific malaria seropositivity and seroconversion rates for participants in Langkat district, North Sumatera Province, Indonesia.

Seroprevalence, % (n/N)									
Category	PfAMA-1	PfMSP-1_19_	p-value^a^	PvAMA-1	PvMSP-1_19_	p-value^a^	PfAMA-1/PfMSP-1_19_	PvAMA-1/PvMSP-1_19_	p-value^b^
≤15	13.3 (11/83)	16.9 (14/83)	0.665	16.9 (14/83)	4.8 (4/83)	0.022	25.3 (21/83)	18.1 (15/83)	0.347
16 to 30	10.9 (5/46)	8.7 (4/46)	0.739	17.4 (8/46)	8.7 (4/46)	0.354	17.4 (8/46)	19.6 (9/46)	0.999
31 to 45	10.3 (8/78)	12.8 (10/78)	0.803	21.8 (17/78)	6.4 (5/78)	0.010	19.2 (15/78)	23.1 (18/78)	0.695
>45	8.3 (11/132)	12.1 (16/132)	0.417	18.2 (24/132)	9.1 (12/132)	0.047	19.7 (26/132)	21.2 (28/132)	0.879
All ages	10.6 (36/339)	13 (44/339)	0.405	18.6 (63/339)	7.4 (25/339)	<0.001	20.7 (70/339)	20.7 (70/339)	0.999
SCR (λ)^c^ (95% CI)	0.089 (0.000-16.957)	0.053(0.006-0.449)		0.044 (0.009-0.223)	0.008 (0.002-0.033)		0.131 (0.011-1.683)	0.045 (0.011-0.181)	

CI, Confident interval; SCR (λ), Seroconversion rate.

a Comparing individual seroprevalence between *P. falciparum* and *P. vivax* species-specific antigens.

b Comparing combination seroprevalence between *P. falciparum* and *P. vivax* species-specific antigens.

c Data from infants under 1 year of age were excluded from the reversible catalytic model to remove any influence of maternally derived antibodies.

### Transmission intensity and factors associated with transmission

**Figure 2:**
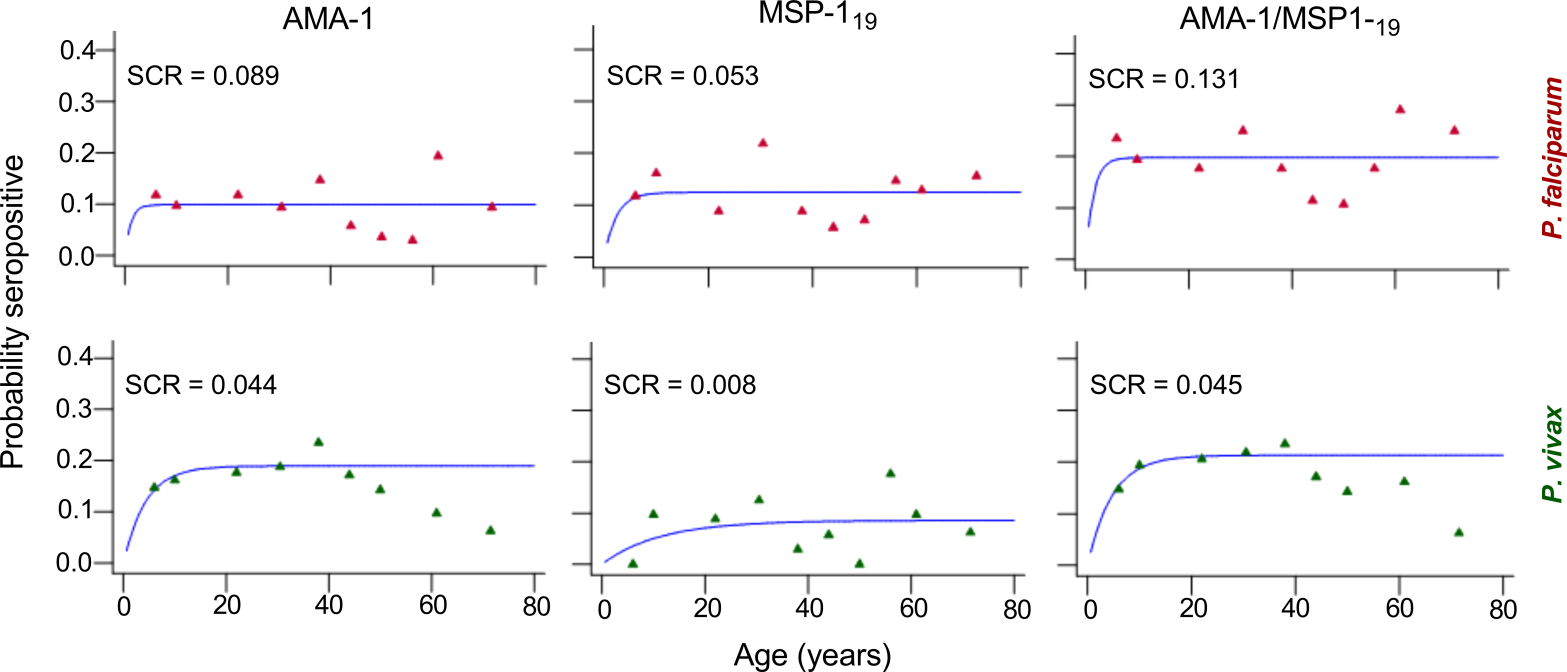
Annual probability of seroconversion rate (SCR) for specific malaria antigen by age in the community of Langkat district, North Sumatera Province, Indonesia. Seropositive data were obtained using age deciles and fitted to reversible catalytic seroconversion models. Points show the observed values within each age group for *P. falciparum* (red) and *P. vivax* (green) recombinant antigens and the blue line shows the fitted curve.

The relationship between seroprevalence and age was further examined using reversible catalytic conversion models. The SCR rates for parasite antigens are shown in **Table 2**; **Figure 2**. Based on the reversible catalytic model, the calculated SCRs indicated a higher level of *P. falciparum* transmission than *P. vivax* using all tested antigens. The *P. falciparum* SCR was 0.089 person-year (95% CI: 0.000–19.957), 0.053 (95% CI: 0.006–0.449) and 0.131 person-year (95% CI: 0.011–1.683) for PfAMA-1, PfMSP-1_19_ and PfAMA-1/PfMSP-1_19_, respectively. The *P. vivax* SCR was 0.053 person-year (95% CI: 0.006–0.449), 0.008 (95% C: 0.002-0.033) and 0.045 person-year (95% CI: 0.011–0.181) for PvAMA-1, PvMSP-1_19_ and PvAMA-1/PvMSP-1_19_, respectively. Nevertheless, the SCRs were not statistically significant between species-specific antigens, evidenced by the overlapping confidence intervals. Univariate and multivariate logistic regression analyses to identify factors associated with seropositivity to a combination of any *P. falciparum*– and *P. vivax*–specific antigens are shown in **Table 3** and **Table 4**, respectively. Only spending nights in the forest was associated with *P. vivax* seropositivity in the adjusted model. The crude odd ratio (OR) of *P. vivax* seropositivity for those spending nights in the forest compared to those who are not was 3.89 (95% CI: 2.25–6.75, p < 0.001). The increased trend for *P. vivax* positivity remained apparent in the adjusted model (AOR: 3.93, 95% CI: 2.21–7.14, p < 0.001). For *P. falciparum*, no variables were significantly associated with seropositivity in the adjusted model (all p > 0.05).

**Table 3:** Logistic regression analysis of explanatory factors for serological evidence of exposure to *P. falciparum* in Langkat district, North Sumatera Province, Indonesia.

Category	Seropositive, % (n/N)	COR (95 % CI)	p-value	AOR (95 % CI)	p-value
Gender					
Male	22.2 (24/108)	1		1	
Female	19.9 (46/231)	0.87 (0.49-1.52)	0.625	0.95 (0.53-1.69)	0.861
Age group					
≤15	25.3 (21/83)	1		1	
16–30	17.4 (8/46)	0.62 (0.25-1.54)	0.305	0.43 (0.13-1.41)	0.162
31–45	19.2 (15/78)	0.71 (0.33-1.49)	0.357	0.47 (0.16-1.39)	0.173
>45	19.7 (26/132)	0.72 (0.38-1.39)	0.334	0.48 (0.17-1.36)	0.169
ITN ownership					
No	19.6 (46/235)	1		1	
Yes	23.1 (24/104)	1.23 (0.71-2.15)	0.463	1.05 (0.41-2.64)	0.924
ITN use					
No	19.4 (38/196)	1		1	
Yes	22.4 (32/143)	1.19 (0.71-2.03)	0.502	1.15 (0.48-2.73)	0.756
Occupation					
Non-forest goers	9.1 (2.22)	1		1	
Forest goers	21.1 (42/199)	2.68 (0.61-11.91)	0.196	2.56 (0.57-11.49)	0.220
Unemployed	22 (26/118)	2.83 (0.62-12.89)	0.180	2.27 (0.47-11.06)	0.309
Live within forest fringe					
No	23.4 (11/47)	1		1	
Yes	20.2 (59/292)	0.83 (0.39-1.72)	0.615	0.76 (0.35-1.68)	0.498
Spend night at the forest					
No	19.3 (42/218)	1		1	
Yes	23.1 (28/121)	1.26 (0.74-2.17)	0.399	1.35 (0.75-2.41)	0.314
History of malaria					
No	20.5 (69/337)	1		1	
Yes	50 (1/2)	3.88 (0.24-62.88)	0.340	4.68 (0.27-80.31)	0.287

AOR, Adjusted odd ratio; COR, Crude odd ratio; CI, Confident interval; ITN, Insecticide-treated net.

**Table 4:** Logistic regression analysis of explanatory factors for serological evidence of exposure to *P. vivax* in Langkat district, North Sumatera Province, Indonesia.

Category	Seropositive, % (n/N)	COR (95 % CI)	p-value	AOR (95 % CI)	p-value
Gender					
Male	21.3 (23/108)	1		1	
Female	20.4 (47/231)	0.94 (0.54-1.65)	0.840	1.07 (0.59-1.95)	0.825
Age group					
≤15	18.1 (15/83)	1		1	
16–30	19.6 (9/46)	1.11 (0.44-2.76)	0.835	0.80 (0.24-0.72)	0.727
31–45	23.1 (18/78)	1.36 (0.63-2.93)	0.433	0.86 (0.28-0.67)	0.791
>45	21.2 (28/132)	1.22 (0.61-2.45)	0.576	0.81 (0/28-2.35)	0.697
ITN ownership					
No	20.9 (49/235)	1		1	
Yes	20.2 (21/104)	0.96 (0.54-1.70)	0.89	0.83 (0.32-2.14)	0.704
ITN use					
No	20.4 (40/196)	1		1	
Yes	20.9 (30/143)	1.04 (0.61-1.76)	0.898	1.16 (0.49-2.78)	0.722
Occupation					
Non-forest goers	13.6 (3/22)	1		1	
Forest goers	23.6 (47/199)	1.96 (0.56-6.91)	0.296	1.76 (0.48-6.51)	0.397
Unemployed	16.9 (20/118)	1.29 (0.35-4.79)	0.701	1.18 (0.29-4.92)	0.814
Live within forest fringe					
No	12.8 (6/47)	1		1	
Yes	21.9 (64/292)	1.92 (0.78-4.72)	0.156	0.95 (0.36-2.51)	0.914
Spend night at the forest					
No	12.4 (27/218)	1		1	
Yes	35.5 (43/121)	3.89 (2.25-6.75)	<0.001	3.93 (2.12-7.14)	<0.001
History of malaria					
No	20.8 (70/337)				
Yes	0 (0/2)				

AOR, Adjusted odd ratio; COR, Crude odd ratio; CI, Confident interval; ITN, Insecticide-treated net.

## Discussion

This cross-sectional study, nested within a prior epidemiological survey on submicroscopic malaria (Nainggolan et al., 2022), analyzed antibody responses to AMA-1 and MSP-1_19_ of *P. falciparum* and *P. vivax* in samples collected from the population in Langkat district, North Sumatera Province, Indonesia. The serological outcomes revealed similarities in the seroprevalence of both species and a relationship between age groups and seroprevalence rates. The study also identified higher transmission levels of *P. falciparum* compared to *P. vivax* based on SCR and found that spending nights in the forest was a risk factor associated with *P. vivax* exposure. These findings could help inform malaria elimination efforts in Indonesia and support the potential integration of seroepidemiological methods into routine elimination programs.

The antigens AMA-1 and MSP-1_19_ were selected because they are present in both species, represent the erythrocytic stage of the parasites, and have been widely used as markers of exposure in the past. The analysis of antibody responses among the study participants revealed a similar seroprevalence rate of 20.7%, with individuals responding to at least one antigen from either *P. falciparum* or *P. vivax*. Although no other serological surveys had been conducted previously in Langkat district, these rates were relatively higher compared to those found in similar epidemiological settings in Sumatera, such as a population survey in Aceh Province, Indonesia, in 2013 (6.9% for *P. falciparum* and 2%, for *P. vivax*) (Surendra et al., 2019). Nevertheless, these findings are inconsistent with reports from other countries with *P. falciparum*–*P. vivax* co-endemicity in the Southeast Asian region where antibodies against *P. falciparum* antigens were found to be higher than *P. vivax* antigens such as Malaysia (58% and 10%) (Rahim et al., 2023), Thailand (79% and 40%) (Rahim et al., 2022), Vietnam (38% and 31%) (San et al., 2022), and Myanmar (30% and 14%) (Edwards et al., 2021). The observed difference in seroprevalence rates could be attributed to the fact that antibody responses reflect cumulative exposure events, including past asymptomatic submicroscopic infections (Idris et al., 2017a; Macalinao et al., 2023), and the relatively long half-life of responses against Pf/PvAMA-1 and Pf/PvMSP-1_19_ antigens generated after exposure (San et al., 2022). Additionally, the historical co-dominance of *P. falciparum* and *P. vivax* in the study area with similar prevalence of 13.8% and 13.6%, respectively (Lubis et al., 2017), may have contributed to the similar seroprevalence rates observed for both species in the population, highlighting the need for targeted malaria elimination strategies in Indonesia.

Seroprevalence reflects cumulative malaria exposure and modelling changes between seroprevalence and age (i.e. SCR), can be used to estimate transmission intensity in a population (Idris et al., 2017a; van den Hoogen and Drakeley, 2017). In this study, SCRs estimated from the age-adjusted seroprevalence curves for *P. falciparum* antigens were higher than the ones for *P. vivax* antigens, reflecting the more intense transmission of the former species in Langkat district. These differences in SCR estimates are likely to reflect the different ecological factors that affect malaria exposure and the acquisition of immunity to malaria in the area (Cunha et al., 2014). Unfortunately, data on the ecology of malaria parasitism in this survey were not recorded to enable the testing of these hypotheses. Additionally, different transmission patterns for falciparum and vivax as evidenced by data are likely to reflect the actual difference in transmission of the two species observed over the years. Furthermore, the higher seroconversion rate of *P. falciparum* compared to *P. vivax* may be due to its more frequent symptomatic infections, higher transmission intensity, and possibly longer persistence of antibodies post-infection (Kattenberg et al., 2018; Herman et al., 2023). *P. falciparum* also typically causes more severe disease, prompting a stronger and more detectable immune response over time (van den Hoogen et al., 2020).

The estimated SCR for the AMA-1 was higher than that of MSP-1_19_ for both *P. falciparum* and *P. vivax* in Langkat District, aligning with results from other seroepidemiological studies (Cook et al., 2010; Tusting et al., 2014; Wong et al., 2014; Idris et al., 2017b; Kwenti et al., 2017; Surendra et al., 2019; van den Hoogen et al., 2020). The differences in transmission estimates between AMA-1 and MSP-1_19_ may be attributed to variations in seroconversion and reversion rates, which are potentially influenced by differences in immunogenicity, subclass-dependent half-life, and antigen polymorphism (Badu et al., 2012). AMA-1, being more immunogenic and associated with higher antibody titers than MSP-1_19_, likely exhibits faster seroconversion and seroreversion rates (Drakeley et al., 2005), which might also explain the observations. Furthermore, the absence of an age-related trend in seroprevalence for the AMA-1 and MSP-1_19_ antigens of both *P. falciparum* and *P. vivax* in the present study limits their value when analyzed using the current modelling approach. Alternative serological estimates of malaria transmission intensity, such as the antibody acquisition model (Yman et al., 2016) and the unified mechanistic model (Kyomuhangi and Giorgi, 2021) can enhance the precision of transmission estimates.

Multivariate regression analyses identified that for *P. falciparum*, no variables were significantly associated with seropositivity in the adjusted model. However, spending nights in the forest was associated with *P. vivax* seropositivity. This observed association could be due to the distinct ecological and behavioral patterns of the vectors that transmit *P. vivax*, which are often more exophagic (outdoor-biting) and exophilic (outdoor-resting) compared to those transmitting *P. falciparum* (Baird, 2013). A recent study in Sumatera showed that predominant *P. vivax* vectors, such as *Anopheles dirus* (Aceh Province) and *Anopheles kochi* (North Sumatera Province), thrive in forested environments where they have greater access to humans sleeping outdoors or in forested areas (Permana et al., 2023), which could lead to higher exposure risk. This is consistent with the reported risk factors from a previous study, where 59.4% of individuals had forest-associated occupation, and 75.7% had a history of forest visits (Nainggolan et al., 2022). In contrast, *P. falciparum* vectors are generally more endophagic (indoor-biting), reducing the likelihood of transmission in forest settings (Cohen et al., 2012). The resilience of *P. vivax* to low-density environments and its ability to persist in temperate climates further increases its transmission potential in forested regions (Battle et al., 2012; Yman et al., 2016). Additionally, *P. vivax* hypnozoites can cause relapses, maintaining seropositivity even with intermittent exposure (Flannery et al., 2022).

This study had several limitations. The most significant was the insufficient sample size, which inevitably reduced the precision of the current SCR estimates (Sepúlveda et al., 2015). Additionally, the disproportionate sampling and overrepresentation of adult participants may have led to results that are not fully representative of the study population. This oversampling likely occurred due to the timing of surveys, as most were conducted on weekdays when children were in school. Furthermore, the study was restricted to a small geographical area in Langkat district, a region with very low malaria endemicity (Lubis et al., 2017; Nainggolan et al., 2022). This limitation raises concerns about the generalizability of the findings to other areas of Indonesia or beyond. Lastly, the study utilized only two recombinant antigens for each *Plasmodium* species. While these antigens are considered long-term markers of transmission (Drakeley et al., 2005), individual variations in immune responses to different parasite antigens suggest that using a broader range of antigens, including short-term markers, might improve the identification of seropositive individuals (Kerkhof et al., 2016). Despite these limitations, the study contributes to the collective body of knowledge on malaria antibodies and lays the groundwork for the potential future use of this tool in Indonesia, which is especially relevant for other countries also aiming for malaria elimination.

## Conclusion

This study assessed antibody responses to AMA-1 and MSP-1_19_ antigens of *P. falciparum* and *P. vivax* in Langkat District, Indonesia, revealing similarities in seroprevalence and significant age-related variations in seropositivity rates. The findings indicate higher transmission levels of *P. falciparum* compared to *P. vivax*, aligning with global patterns but diverging from observations in co-endemic settings in Southeast Asia. The association between spending nights in the forest and increased *P. vivax* seropositivity highlights the role of vector behavior and ecological factors in transmission dynamics. These insights emphasize the need for targeted interventions tailored to specific populations with varying demographics and risk factors, as well as the integration of seroepidemiological tools into malaria elimination strategies in Indonesia. By understanding the distinct transmission patterns and associated risk factors, tailored approaches can be developed to address the specific challenges of *P. falciparum* and *P. vivax* elimination.

## Data Availability

The raw data supporting the conclusions of this article will be made available by the authors, without undue reservation.
